# Dynamic Morphospaces for Animal Locomotion: A Case Study in Bird Flight

**DOI:** 10.1093/icb/icag058

**Published:** 2026-06-04

**Authors:** Lydia A France, Christina Harvey, Graham K Taylor

**Affiliations:** Department of Biology, University of Oxford, South Parks Road, Oxford OX1 3EL, UK; Department of Mechanical and Aerospace Engineering, University of California, Davis, One Shields Ave, Davis, CA 95616, USA; Department of Biology, University of Oxford, South Parks Road, Oxford OX1 3EL, UK; Department of Mechanical and Aerospace Engineering, University of California, Davis, One Shields Ave, Davis, CA 95616, USA

## Abstract

Morphospaces represent the range of possible biological designs by placing different shapes into a common geometric space. They have revealed that high-dimensional morphological diversity in biological systems tends to concentrate along a reduced set of coordinated axes. Here, we argue that extending this framework from static form to dynamic behavior provides a principled way to describe, compare, and translate animal locomotion. Constructing a kinematic morphospace from landmark positions yields a Euclidean space that supports not just dimensionality reduction but reconstruction, projection into shared coordinate systems, and formal comparison of subspaces across individuals and species. Dynamic Mode Decomposition is an additional complementary approach which identifies how shape changes are sequenced in time. Drawing on avian flight as a case study, we review how these tools connect the morphospace tradition in evolutionary biology to emerging questions in locomotor biomechanics, and discuss how the biological morphospace can serve as a common coordinate system for bio-informed engineering design.

## Introduction

While maneuvering through the air, birds coordinate rapid, simultaneous changes in wing and tail geometry and body posture. Traditionally, we document these maneuvers using established metrics, such as joint angles, wingbeat amplitude, and wingspan, that fragment this integrated motion into dimensions chosen by researchers (Dickinson et al. 2000). These approaches have revealed many key findings and opened the door for animal flight research (Vance et al. 1990; Vance 1992; Tobalske and Dial 1996; Carruthers et al. 2010; Altshuler et al. 2012; Berg Robertson and Biewener 2012; Crandell and Tobalske 2015; Usherwood et al. 2020) These metrics are often selected based on expected physics or engineering theory and to align with what is possible to experimentally measure (Hedrick et al. 2012; Ros et al. 2014). The apparent complexity in biological locomotion is real and likely informative (Dudley 2002), but may be exacerbated by the choices of reference frame and measurement variables. By using these established metrics, it is possible that we impose a human-centric view on animal motion, rather than capturing nuances that may be critical for effective locomotion. One example of a related challenge that has puzzled researchers is the search for discrete flight “gaits” within birds (Tobalske and Dial 1996; Hedrick et al. 2002; Tobalske 2007).

Here, we discuss methods for developing a workflow that captures the integrated, coupled and continuous nature of locomotion (Nishikawa et al. 2007), allowing animal movement to be re-evaluated within a framework derived directly from shape and motion. We propose that these methods may allow animal locomotion to be understood in terms of a simpler underlying structure, one that is shared across species and can be translated to inform the design of engineered systems (Harvey et al. 2022, 2023).

Evolutionary biology has encountered a similar challenge associated with human-selected metrics when comparing morphological form across species. Organisms vary as integrated structures, and examining landmark coordinates alone provides little insight into overall shape (Bookstein 1992). Geometric Morphometrics addressed this through methods such as generalized procrustes analysis (GPA), which aligns varying forms for comparison, and principal component analysis (PCA), which identify coordinated axes defined by maximizing shape variation directly from tracked landmark data (Rohlf and Slice 1990; Dryden and Mardia 2016). Morphological variation is often concentrated along a reduced set of coordinated directions, indicating a lower-dimensional structure (Mitteroecker and Gunz 2009; Adams et al. 2013).

**Fig. 1:**
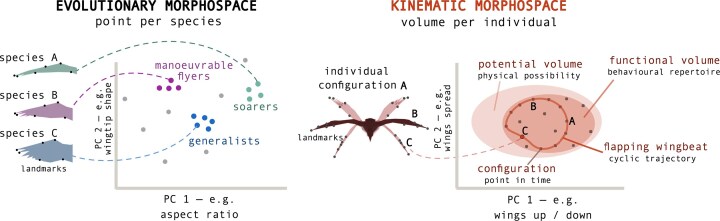
Evolutionary and kinematic morphospaces compared. *Schematic illustration*. Left: in an evolutionary morphospace, each species is represented by a single point derived from specimen landmarks, and axes capture interspecific shape variation (e.g., aspect ratio and wingtip shape). Clusters of species reflect morphological similarities that can be linked to ecological or behavioral traits, such as soaring. Right: in a kinematic morphospace, specimen landmarks are tracked on an individual over time. Each configuration maps to a point in a space defined by coordinated patterns of shape change (e.g., wing sweep, span, or tail configuration). Each individual therefore occupies a volume representing the range of shapes used during behavior, with trajectories through this volume corresponding to sequences of motion. The full behavioral repertoire defines a functional volume nested within a larger potential volume of physically possible configurations, which may not all be explored during typical behavior. Unlike evolutionary morphospaces where species are represented as points, kinematic morphospaces represent behavior as trajectories within a continuous space of coordinated shape change. Morphospaces may have higher dimensionality, but only two axes are shown here for visual clarity.

In flight, coordinated shape change occurs within a single individual over seconds rather than across species over evolutionary time. In this representation, an individual occupies not a single position in morphospace but a volume representing the range of shapes used across its behavioral repertoire. This distinction between evolutionary and kinematic morphospaces is illustrated in Fig. 1, which contrasts evolutionary morphospaces, where each species occupies a point, with kinematic morphospaces, where individuals occupy volumes corresponding to their behavioral repertoire. Methods such as PCA can identify the dominant axes of shape variation that define this space. However, these methods do not account for the timing of shape change, which is critical for aerodynamic function. Dynamic Mode Decomposition (DMD) captures temporal structure by identifying spatiotemporal modes directly from kinematic data (Schmid 2010; Kutz et al. 2016; Demo et al. 2018; Ichinaga et al. 2024).

Here, we review the morphospace tradition in evolutionary biology and connect it to its emerging role in behavioral analysis, drawing on recent analyses in avian flight kinematics (France et al. 2025, 2026). We discuss how these reduced representations can inform bio-informed engineering design and control by providing a principled, reduced-dimensional representation of the feasible design space within an otherwise vast set of possibilities.

## Morphospaces in evolutionary biology

The power of morphospace analysis in evolutionary biology lies in a shift in perspective: rather than examining individual measurements in isolation, the approach represents whole organisms as points in a multidimensional space defined by shared variation. In a morphospace, each organism occupies a position, distances between points reflect phenotypic differences, and covariation structure can be analyzed quantitatively to compare shapes and evolutionary patterns across taxa (Mitteroecker and Huttegger 2009).

In Geometric Morphometrics, homologous landmarks are placed on each specimen, and methods such as GPA remove differences in position, orientation, and scale to align specimens into a common coordinate system (Gower 1975; Rohlf and Slice 1990). PCA of these aligned coordinates identifies the directions in which landmarks change together, producing a reduced set of coordinated axes that capture the dominant patterns of shape variation in the sample (Jolliffe 2002; Zelditch et al. 2004).

The resulting morphospace provides an intuitive and quantitative framework in which evolutionary change can be visualized as trajectories, and patterns of morphological variation and disparity can be characterized and compared across clades and time (Mitteroecker and Gunz 2009). This framework has been transformative because biological variation in form often concentrates along a limited set of coordinated axes rather than being uniformly distributed across all high-dimensional variables, revealing lower-dimensional structure in complex morphological data (Cheverud 1982; Wagner 1984; Hallgrímsson et al. 2009).

This framework has been applied widely across diverse clades and anatomical structures. For example, geometric morphometric analyses of avian wing shape across hundreds of species show that variation in planform tends to align along axes associated with aspect ratio and wingtip geometry, and that ecological differences among species align with these major morphological gradients (Sheard et al. 2020; Rader and Hedrick 2023). Similarly, skull morphospaces constructed from homologous cranial landmarks have been used to characterize broad patterns of cranial diversity in mammals, reptiles, and fishes, revealing that much of the variation can be described by a reduced set of axes capturing size-related allometry and putative functional trade-offs in feeding and sensory systems (Stayton 2005; Sicuro and Oliveira 2011; Bright et al. 2016; Felice and Goswami 2018; Evans et al. 2019). In insects, morphospaces based on wing shape and venation patterns have been used to compare morphological diversity across orders, quantifying variation in wing outline, vein topology, and other structural features that relate to flight mechanics and evolutionary divergence (Wootton 1992; Perrard et al. 2014). Across these and other systems, the recurring finding is that high-dimensional morphological variation, whether in wings, skulls, or body plans, tends to concentrate along a reduced-dimensional subspace defined by coordinated patterns of shape change, a phenomenon widely documented in geometric morphometric studies and reviews of the field (Polly and Motz 2016). In many biological systems, high-dimensional phenotypes tend to be constrained to a lower-dimensional space of variation by developmental and genetic integration, so that variation and evolutionary change concentrate along a limited number of dominant directions in morphospace (Olson and Miller 1999; Armbruster et al. 2014).

Extending this framework from static forms to morphing shapes used in locomotion is a natural step: the same Geometric Morphometrics methodology can be applied to landmarks as they move during behavior rather than through evolutionary history. This is a promising approach as there is reason to expect a reduced dimensional structure in kinematics. Similar low-dimensional structure has been observed across model locomotor systems, where a limited set of coordinated postures captures the majority of behavioral variation (Hassinan et al. 2024). Bernshteĭn (1967) recognized that the musculoskeletal system has far more possible configurations than any single task requires, and proposed that biological systems resolve this redundancy by coupling degrees of freedom into coordinated groupings (Bernshteĭn 1967; Latash 2012). If evolutionary morphospaces exhibit reduced dimensional structure due to developmental and genetic integration, kinematic morphospaces may exhibit similar structure due to neuromuscular coupling.

## Kinematic morphospaces

A kinematic morphospace represents each instantaneous posture as a point in a space defined by coordinated patterns of shape change (as illustrated in Fig. 1). Rather than following individual joint angles separately, this approach captures how multiple parts of the body deform together during locomotion. PCA has been widely used to identify the main axes of coordinated shape change during motion (Troje and Troje 2002; Daffertshofer et al. 2004; Riskin et al. 2008; Stephens et al. 2008; Groves-Raines et al. 2024). In PCA, the orthogonal deformation axes are chosen to maximize variance and span a reduced-dimensional linear subspace of the full landmark configuration space. These axes are statistical descriptions of coordinated variation and do not correspond directly to physical actuation or control variables. Once these axes are defined, each posture can be represented by its coordinates along them. As in geometric morphometrics, configurations can be reconstructed from these coordinates by combining the average shape with weighted contributions from each axis, which ensures that the reduced representation remains anatomically interpretable: every point in morphospace corresponds to a physically realizable landmark configuration (Zelditch et al. 2004; Dryden and Mardia 2016).

When kinematic data are projected into morphospace, each configuration at a given time is represented by coordinates along the principal axes. As the movement unfolds, these coordinates vary through time, forming a trajectory in morphospace that describes how different coordinated patterns of shape change combine across the movement. In this way, a complex movement can be interpreted as the evolving combination of a limited set of shape change patterns, while full landmark configurations can still be reconstructed at any frame. By separating coordinated patterns of variation, this representation can make smaller deformations more visible when they would otherwise be masked by larger motions, such as fine-scale hand-wing adjustments during flapping or coordinated changes in wing shape during takeoff, as observed in previous studies (France et al. 2025; Huang et al. 2025).

Dimensionality reduction provides several complementary ways to assess and compare kinematic structure. First, dimensionality reduction provides a basis for assessing how well the reduced representation captures the original data. Landmark configurations can be reconstructed from a subset of axes, and the ratio of variance explained by those axes quantifies how well the reduced representation approximates the full data (Adams et al. 2013; Dryden and Mardia 2016). Because PCA finds the subspace that minimizes reconstruction error for a given number of axes, reconstruction error decreases systematically as additional axes are included, allowing the dimensional structure of the morphospace to be chosen explicitly rather than assumed.

Second, a PCA basis derived from one dataset can be used to assess how well it represents another. New configurations can be expressed in the reference PCA space and reconstructed back into landmark positions. The difference between the reconstructed and original configurations, quantified as root-mean-square error, provides a straightforward measure of how well the dominant patterns of shape change in one dataset generalize to another. For example, previous work in avian flight kinematics demonstrates how such cross-dataset reconstruction can be used to compare coordinated patterns of motion across individuals (France et al. 2025).

While reconstruction within a single dataset is standard practice in geometric morphometrics, using cross-dataset reconstruction fidelity in this way offers a quantitative basis for comparing coordinated kinematics across individuals or conditions. Similar ideas are well established in the statistical shape modeling literature, where PCA-based models are trained on a reference sample and evaluated by their ability to reconstruct previously unseen shapes (Cootes et al. 1995, 2001). It is important to note that principal component axes are specific to the dataset from which they are computed. Directly comparing axes (principal components) or their coordinates across independently derived spaces can therefore be misleading. Meaningful comparison requires projection into a shared reference space or explicit alignment of the corresponding subspaces.

Finally, the spaces themselves can be compared. Techniques such as principal angle analysis provide quantitative measures of similarity between subspaces and are well established in multivariate analysis (Björck and Golub 1973; Mitteroecker and Gunz 2009). Together, these approaches provide a framework for comparing coordinated patterns of locomotion across datasets at multiple levels, from individual configurations to the structure of the underlying spaces.

These methods offer a practical way to compare coordinated patterns of locomotion across individuals, conditions, species, and potentially engineered morphing systems projected into a common biological space (Daffertshofer et al. 2004; Riskin et al. 2008; Federolf et al. 2013; Skandalis et al. 2024; France et al. 2025). However, shifting from evolutionary to behavioral timescales raises additional considerations, including the choice of coordinate representation and the role of temporal structure in constructing and interpreting kinematic morphospaces.

## Coordinate representation in kinematic morphospace

In biomechanics, PCA is frequently applied to joint angle measurements and landmark coordinates for dimensionality reduction, yielding valuable insights into dominant patterns of joint coordination during specific behaviors (Daffertshofer et al. 2004; Riskin et al. 2008; Altshuler et al. 2012; Federolf et al. 2013; Huang et al. 2025). However, the geometric structure of the coordinate system influences how the resulting statistics should be interpreted.

PCA is defined in a Euclidean vector space: a flat space in which distances, linear combinations, orthogonality, and covariance are well defined (e.g., the familiar Cartesian coordinates $[x, y, z]$). In such spaces, averages lie along straight-line segments and variance-covariance can be summarized through linear projections. This assumption underlies the application of PCA and other linear methods to kinematic data.

**Fig. 2:**
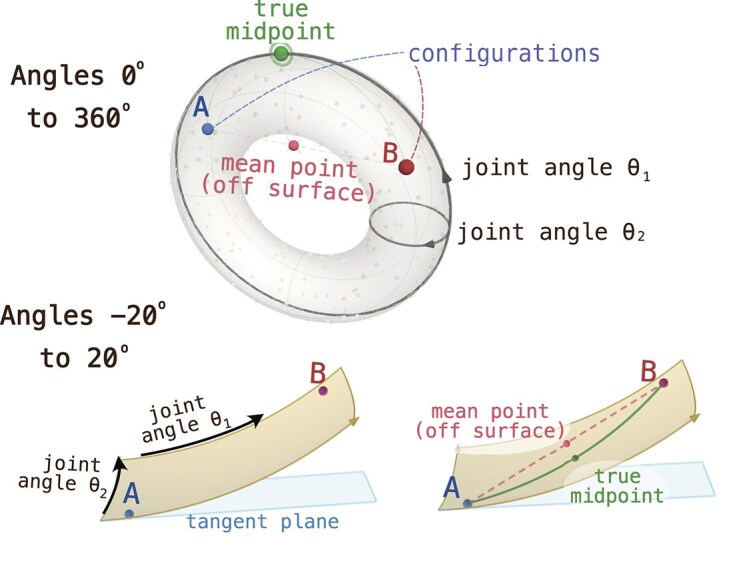
Geometric considerations for coordinate representation in morphospace. Joint angles are periodic variables, meaning that values such as $0^\circ$ and $360^\circ$ represent the same configuration. This creates a curved configuration space rather than a flat one. Top: when angles span their full range ($0^\circ$–$360^\circ$), two configurations (*A, B*) lie on the surface of a torus. Their true midpoint also lies on this surface, but the simple arithmetic mean is off-surface, corresponding to a posture that is not physically realizable. Bottom: restricting the angular range (e.g., $-20^\circ$ to $20^\circ$) makes the space locally more linear (approximated by a tangent plane), so averages become more meaningful. However, the underlying curvature remains, and the computed mean still deviates from the true midpoint on the surface. This has important statistical consequences, as linear methods such as PCA rely on operations (e.g., averaging and covariance) that may not be valid in curved configuration spaces.

Joint angles, however, are periodic variables (e.g., $0^\circ$ and $360^\circ$ represent the same configuration). When multiple rotational degrees of freedom are combined, they define a curved configuration space rather than a flat vector space (Fig. 2). For example, two freely rotating joints define a torus. On such spaces, straight-line interpolation or arithmetic averaging of angles does not necessarily correspond to meaningful intermediate postures, because the space is curved and topologically circular. An arithmetic average between two poses may lie off the surface entirely, corresponding to a configuration that is not physically attainable. These features arise from the curvature and periodic topology of the configuration space. This geometric distinction does not preclude the use of linear methods, but they operate as approximations to an underlying curved space. As a result, care is required when applying linear methods such as PCA to joint angle data, particularly when angles span large ranges.

Angular variables can also be embedded in Euclidean space, for example, by representing each angle using sine and cosine components, which removes discontinuities associated with periodicity. However, this embedding increases the dimensionality of each rotational degree of freedom and constrains the data to lie on a unit circle within the embedding space. Linear combinations of these coordinates do not automatically remain on that circle without additional normalization, and principal components therefore become combinations of sine and cosine components that may be less directly interpretable as simple joint rotations.

Restricting angular range can reduce discontinuities and improve local linear approximation. However, curvature effects persist even over relatively small angular ranges, and the underlying configuration space remains non-Euclidean. As a result, linear methods such as PCA should be interpreted as approximations, with distances and averages evaluated within a linear geometry. In practice, linear methods such as PCA should be interpreted as approximations when applied to angular data, as curvature effects persist even over relatively small angular ranges. This approximation is most useful when the dominant patterns of variation are large relative to the geometric distortion introduced by curvature (rather than when angular variation falls below a specific threshold). Similar considerations apply to other statistical approaches that rely on linear combinations, covariance structure, or Euclidean distance measures, including regression, multivariate analyses of variance, and distance-based clustering.

Robotics and control theory routinely represent motion in curved configuration spaces using tools from differential geometry (Murray et al. 1994). For the comparative and interpretive aims pursued here, however, representing locomotion configurations in Euclidean space offers practical advantages: distances, projections, and averages all have straightforward meanings, and standard linear decompositions apply directly without requiring more specialized geometric tools. This choice reflects a trade-off between geometric fidelity and interpretability, prioritizing representations that are straightforward to analyse and compare across datasets.

Representing posture using body-centered landmark coordinates provides such a Euclidean space, with globally interpretable distances and directions. In this sense, the framework parallels evolutionary morphospaces; configurations become points in a shared geometric space defined by coordinated variation.

However, extending the morphospace concept from evolutionary form to locomotor behavior involves different geometric assumptions. In classical evolutionary morphometrics, landmark configurations are aligned using Procrustes superimposition to remove differences in position, orientation, and scale, isolating shape from pose. This approach is appropriate when orientation and size are uninformative variables relative to the shape of interest. In locomotion, however, global orientation and relative magnitude of movement are often functionally meaningful. Changes in pitch, roll, wing sweep, or banking are integral to maneuvering and force production rather than incidental variation. For these reasons, kinematic configurations can be represented in a body-centered coordinate frame that removes translation while preserving orientation and spatial relationships among landmarks. This representation removes global translation of the body through the fluid to isolate intrinsic shape change from external motion. While this separation is useful for analysing coordinated deformation, global motion remains important for interpreting aerodynamic outcomes and can be analysed alongside the kinematic representation when required.

Similarly, landmark coordinates are often preserved in their natural spatial scale rather than standardized to equal variance. Rescaling or Z-scoring is common and often considered best practice when applying PCA to variables with different units and scales. In many statistical contexts, variables are normalized to prevent differences in numerical magnitude from dominating the analysis. In locomotion, spatial magnitude itself carries biomechanical significance, for example, a displacement at the wingtip has different aerodynamic consequences from an equivalent displacement near the wing root. Preserving relative variance allows the dominant axes of variation to reflect physically meaningful coordination patterns rather than artifacts of rescaling. In practice, this representation can be combined with scaling across individuals (e.g., normalizing by a characteristic length such as wingspan) while preserving relative variance within each individual, allowing comparisons across individuals without removing physically meaningful differences in spatial magnitude within a given motion.

Representing posture in body-centered landmark coordinates, therefore, defines a Euclidean space with globally interpretable distances and directions. The purpose of this representation is not to imply that locomotion is intrinsically linear, but to provide a coordinate system in which dominant patterns of coordinated shape change can be described compactly and compared systematically. Joint angles are computed from the same positional relationships among landmarks, so this representation does not discard skeletal information. Any configuration expressed in morphospace coordinates can be reconstructed in full landmark form, from which joint angles and other biomechanical variables may be derived.

### Examples from bird flight

When applied to high-speed motion capture data from free-flying Harris’s hawks (*Parabuteo unicinctus*), PCA on body-centered landmark positions suggests that the majority of variation in morphing is captured by a limited set of modes, indicating a structured organization of morphing flight (France et al. 2025). The dominant axes appear to correlate with coherent changes in wing sweep, span, and tail shape, but emerge from the data without predefined human-selected variables. However, these axes should not be interpreted as literal actuation modes. They are emergent statistical descriptions of how many parts move together, not direct representations of neural or musculoskeletal actions. The observed behaviors can be expressed as time-varying combinations of these modes rather than as activation of any single axis, making the small kinematic differences between turning and straight flights more visible.

Previous analyses of hawk flight suggest that the dominant deformation subspace is largely conserved across individuals performing diverse maneuvers (France et al. 2025). Flights from different individuals can be projected into the same morphospace, and differences between individuals appear in how they occupy the shared subspace: through greater use of a particular deformation axis, or through distinct temporal sequencing of shared modes. Notably, behavior does not appear to cluster into discrete gaits or flight modes within this space. The occupied region is continuous; flights traditionally labeled as “flapping,” “gliding,” or “turning” do not form separable clusters, but instead occupy regions within a smoothly varying space. This is consistent with previous work suggesting that variation in avian flight kinematics is often continuous rather than forming clearly separable categories (Tobalske and Dial 1996; Hedrick et al. 2002; Tobalske 2007).

## Dynamics: changing shape over time

A single configuration does not generate thrust, enable weaving flight between trees, or complete a landing. Locomotion is defined not only by which shapes are used, but by how those shapes are sequenced over time. In morphospace, a movement appears as a trajectory traced through a space of coordinated shape variation. PCA defines the geometric structure of morphospace by identifying coordinated patterns of shape variation, but it is insensitive to the temporal ordering of configurations. Shuffling the time sequence of frames does not change the principal axes.

Temporal structure in biomechanics is often analyzed using frequency-based or cycle-resolved approaches applied to predefined kinematic variables (e.g., stroke amplitude, wingtip path, or joint angles), as in studies of wingbeat kinematics in bird flight (Altshuler et al. 2012; Berg Robertson and Biewener 2012; Taylor et al. 2017; Chin and Lentink 2022). These approaches decompose time series into constituent frequencies and are effective at identifying dominant oscillations such as wingbeat frequency. However, Fourier analysis operates on individual signals and does not directly identify which spatial components move together at a given frequency, and therefore does not directly capture coordinated deformation patterns across the full body. Moreover, Fourier methods assume stationarity and periodicity, making them less suited to transient behaviors such as turning, landing, or maneuver transitions. This motivates approaches that explicitly link spatial coordination with temporal evolution.

**Fig. 3:**
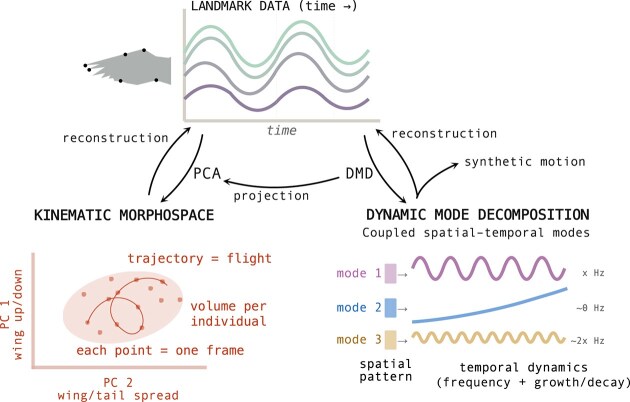
Two complementary decompositions of landmark kinematic data. *Schematic illustration*. Top: Landmark relative positions over time from a moving wing sequence. Left: PCA identifies static axes ranked by variance, defining a kinematic morphospace in which each frame is a point, and each flight traces a path. Configurations can be reconstructed from morphospace coordinates back into landmark positions. Right: DMD identifies coupled spatiotemporal modes from the sequence. Each mode consisting of a spatial pattern paired with a temporal signature (frequency and growth/decay rate). When summed, an approximate of the original landmark motion is reconstructed. Both decompositions operate on the same landmark data and support reconstruction, but PCA captures spatial structure while DMD captures dynamical organization. DMD modes can be projected into PCA space or landmark space, and their dynamics altered to produce synthetic motion such as a wingbeat cycle without decay.

DMD addresses this limitation by linking spatial coordination and temporal evolution within a single framework (Kutz et al. 2016). Rather than analyzing variance alone, or decomposing signals purely by frequency, DMD approximates the kinematics as a system evolving forward in time and identifies coherent spatiotemporal patterns that describe this evolution. This contrasts the spatial decomposition provided by PCA with the spatiotemporal modes identified by DMD. The relationship between spatial and spatiotemporal decomposition is illustrated in Fig. 3, which conceptually contrasts PCA and DMD applied to the same dataset. Similar spatiotemporal structure has been observed in studies of wing deformation, where a small set of shape variables captures spatial variation while their temporal evolution reflects multiples of the wingbeat frequency (Skandalis et al. 2024). DMD was originally developed in fluid mechanics as a tool for identifying coherent structures in high-resolution flow data (Schmid 2010). Early applications focused on systems with relatively clean measurements and well-defined periodic structures. Biological kinematic data, by contrast, are typically noisier, shorter in duration, and less strictly periodic. For this reason, applying DMD to locomotion requires attention to sampling, model order, and robustness. More recent refinements, including optimized and resampled variants of DMD, have improved stability under these conditions, making the approach more suitable for behavioral datasets (Demo et al. 2018; Ichinaga et al. 2024).

In this way, DMD provides a compact representation of how coordinated patterns of shape change evolve over time (Kutz et al. 2016). Each DMD mode extracted from kinematic data consists of a spatial pattern paired with a temporal signature, indicating which landmarks move together at a given frequency and growth or decay rate. In contrast to PCA axes, which are orthogonal directions of maximal variance, DMD modes are eigenvectors of a linear operator approximating the system dynamics, and are not constrained to be orthogonal. They describe how coordinated patterns of motion unfold, persist, or attenuate (Sashidhar and Kutz 2022).

PCA and DMD are complementary methods that extract different structure from the same landmark space. PCA identifies orthogonal axes that capture dominant spatial variation, defining the geometry of morphospace. DMD, by contrast, identifies the dominant dynamical organization of those changes through time. In this sense, DMD complements PCA by linking spatial coordination with temporal evolution within a single decomposition. Because both decompositions are defined in the same underlying landmark configuration space, DMD modes can be expressed by projection in morphospace coordinates, allowing spatial structure and temporal structure to be interpreted within a common geometric framework.

In DMD, the observed kinematics are approximated as a linear combination of these modes. At any given time, the posture is represented as the sum of multiple coordinated patterns of motion, each weighted by a time-dependent amplitude. DMD analysis of hawk flight suggests a limited set of coherent modes that persist across individuals and maneuvers (France et al. 2026). The modes include an oscillatory component corresponding to the wingbeat cycle, slower modes associated with span increase and body rotations, and higher-frequency components localized to the distal wing. These modes provide a compact representation of locomotion as a combination of coordinated spatial patterns evolving on distinct temporal scales (Kutz et al. 2016).

The coexistence of multiple modes suggests that morphing flight may be organized as overlapping dynamical components rather than as discrete states (Skandalis et al. 2024; France et al. 2026). Together, these observations suggest that locomotion may be organized as a set of coordinated patterns evolving on distinct temporal scales. Oscillatory patterns such as wingbeat, slower adjustments such as span increases, and asymmetric corrections during turning can operate simultaneously, each contributing to the overall movement (Tobalske and Dial 1996; Altshuler et al. 2012). Gliding does not emerge as a distinct dynamical state; instead, oscillatory modes decay while lower-frequency patterns dominate. The hawk’s behavior is shaped by changes in the relative amplitude and timing of these modes, rather than by abrupt transitions between qualitatively distinct regimes.

Beyond this, DMD provides a parsimonious representation of locomotion by capturing coordinated spatial and temporal structure in a limited set of modes. These modes and their associated temporal parameters can reconstruct complex kinematic sequences that include multiple maneuvers with low reconstruction error, as indicated in previous work (France et al. 2026). As spatial structure and temporal dynamics are explicitly separated, the temporal properties can be altered while preserving spatial coordination. Frequencies may be adjusted, growth rates modified, or individual modes suppressed to examine their contribution. The resulting model is a compressed recording of movement as well as a structured and manipulable model representation of behavior.

It could be that animal locomotion more generally is also organized both spatially and dynamically in a reduced-dimensional structure (Hassinan et al. 2024). For morphing flight in hawks, and in preliminary analyses of kestrels and pigeons (Proe, et al. 2026), both PCA and DMD suggest that coordinated shape change is structured alongside the temporal sequencing of those patterns. Taken together, spatial morphospace and temporal mode decomposition provide a unified description of locomotor behavior in which geometry and dynamics are treated as complementary aspects of the same system.

DMD also has important limitations. It approximates the observed motion with a linear dynamical model and is therefore best interpreted as a local description of behavior over a particular time window. Strongly nonlinear transitions or highly nonstationary behaviors may not be captured faithfully by a small set of modes. The decomposition can also be sensitive to the number of modes retained and to noise in the data, leading to variation in the identified structure if model order is changed. In this sense, DMD provides a structured summary of a specific sequence rather than a fully parameterized generative model of locomotion. While DMD captures coherent dynamical structure within a given dataset, it does not by itself provide a global parameterization of behavior across conditions. Unlike PCA, which estimates a covariance structure that typically benefits from larger pooled datasets, DMD approximates the dynamics of a specific time-evolution process. It assumes that successive frames are related by a consistent underlying dynamical mapping. Pooling multiple trajectories that differ substantially in behavior, speed, or control strategy may therefore violate this assumption, because the method will attempt to fit a single dynamical model to what may in fact be several distinct regimes. Modes extracted from one flight sequence do not automatically define a continuous coordinate system spanning other maneuvers. This reflects the fact that PCA captures the spatial envelope of configurations, whereas DMD captures the temporal organization of specific trajectories within that space. A different approach or additional modeling alignment would be required to quantify comparisons of modal structure across speeds, environments, or individuals.

## Translating biological structure into engineered systems

A bird that flies successfully within its morphospace demonstrates that the configurations in that space are aerodynamically functional. For engineering, this is valuable because it defines an envelope of functionally validated configurations within an otherwise vast design space. Traditional biomimetic approaches often aim to replicate the biological skeleton’s many degrees of freedom, but this is usually undesirable in aerial vehicles where every mechanical joint adds weight and failure modes. For this reason, we suggest a more bio-informed approach that is explicit about the biological structure from which it draws inspiration (Harvey et al. 2023; Harvey 2026).

**Fig. 4:**
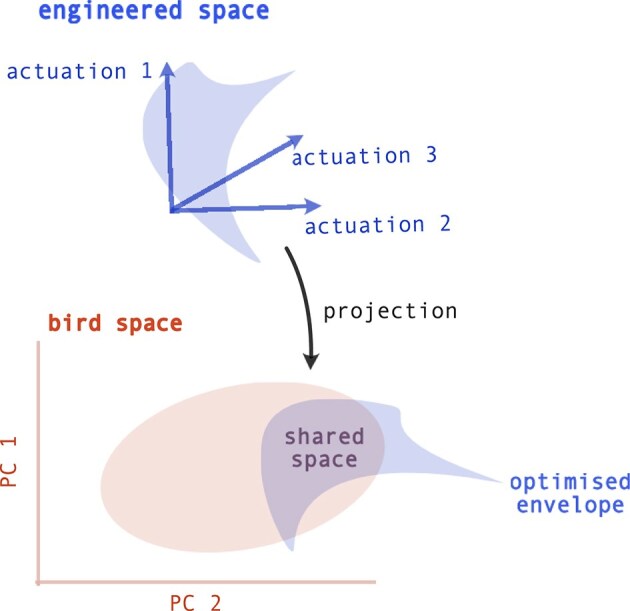
Translating biological morphospace structure into engineered design. *Schematic illustration*. Top: An engineered morphing system has its own actuation degrees of freedom, whose axes need not correspond to the biological axes. Bottom: The biological kinematic morphospace axes represent statistical patterns of coordinated deformation rather than direct representations of muscles, joints, or control inputs. Within the space is a volume of functional repertoire. By projecting the engineered mechanism’s reachable configurations (blue) into the biological morphospace, the two systems can be compared in a shared space. An optimizer can then extend the design envelope beyond configurations explored by the animal (blue, bottom right)

The morphospace framework suggests a different strategy. Rather than replicating biological joints, an engineer designs a mechanism with its own actuatable degrees of freedom—hinges, compliant elements, or morphing skins—chosen for manufacturability and reliability. The PCA and DMD modes are statistical descriptions of coordinated biological deformation, not actuation blueprints; there is no reason a single actuator should produce motion aligned with a single principal component. In this sense, the biological modes define a coordination structure rather than a control architecture. An engineered mechanism will therefore have its own set of actuation axes, potentially more numerous and differently oriented than the biological modes. What matters is whether the mechanism’s reachable configurations, when projected into the biological morphospace, cover the functionally relevant region, with their temporal organization aligning with the spatiotemporal demands of the task. The morphospace provides the common coordinate system for this comparison: biological and engineered systems can be assessed side by side regardless of differences in their physical implementation. This translation from biological to engineered design space is illustrated in Fig. 4.

Crucially, the engineered design need not remain within the region the bird occupies. The biological morphospace provides a principled starting point and proof of functional relevance, but an optimizer initialized in this space can use simulation tools, such as aerodynamic analysis, to push beyond the biological envelope toward configurations that no animal explores (Wong et al. 2026). DMD adds temporal information to this picture by separating the wingbeat from slower morphing dynamics and identifying which coordinated deformations are relevant to functions such as flap-to-glide transitions or turning, as well as the frequencies at which they operate. These provide additional design targets–not just what shapes to produce but how to sequence them in time.

Like all methods, this framework has limitations. One is that data reduction techniques often assume that small changes in shape contained within higher-order dimensional states are negligible for function. While often reasonable assumption at times, this does not hold in all situations and may be especially important when considering aerodynamic function. For example, small changes in the wing angle of attack can cause large changes to the resultant forces and moments produced in flight (Thomas and Taylor 2001).

Much remains open. Whether kinematic similarity reliably predicts aerodynamic performance, how to parametrize smoothly across flight conditions, and whether coordinate systems that are convenient for description reflect those used by the nervous system for control are all empirical questions. If locomotion, like morphology, concentrates along a reduced set of coordinated directions when measured in the right coordinates, this suggests that the right representations can make complex behavior tractable, comparable, and ultimately translatable.

## Conclusion

Morphospaces have transformed how evolutionary biologists understand morphological diversity, revealing that high-dimensional variation often concentrates along a reduced set of coordinated axes. Extending this framework to locomotion suggests that the same principle may hold for behavior: the integrated shape changes that animals use in movement exhibit reduced-dimensional structure, are continuous, and often shared across individuals. Temporal decomposition through methods such as DMD complements this spatial picture by identifying the coherent oscillatory patterns that sequence shape change in time. Together, these approaches provide a compact and principled description of locomotor behavior that can inform engineering design and control.

## Data Availability

No new data were generated or analysed in support of this review.

## Notes

From the symposium “Dinosaurs to Drones: Comparative Approaches to Understanding Flight” presented at the annual meeting of the Society for Integrative and Comparative Biology, January 3–7th, 2026.
